# Subcellular Reactive Oxygen Species (ROS) in Cardiovascular Pathophysiology

**DOI:** 10.3390/antiox7010014

**Published:** 2018-01-16

**Authors:** Sarah Aldosari, Maan Awad, Elizabeth O. Harrington, Frank W. Sellke, M. Ruhul Abid

**Affiliations:** 1 Cardiovascular Research Center, Division of Cardiothoracic Surgery, Department of Surgery, Rhode Island Hospital, Brown University Alpert Medical School, 1 Hoppin St, Coro West 5.231, Providence, RI 02903, USA; sraldosari@alfaisal.edu (S.A.); Mawad@alfaisal.edu (M.A.); fsellke@lifespan.org (F.W.S.); 2 Providence VA Medical Center, Providence, RI 02908, USA; elizabeth_harrington@brown.edu

**Keywords:** ROS, cardiovascular disease, Nicotinamide Adenine Dinucleotide Phosphate (NADPH) oxidase, mitochondrial ROS, angiogenesis, coronary endothelium

## Abstract

There exist two opposing perspectives regarding reactive oxygen species (ROS) and their roles in angiogenesis and cardiovascular system, one that favors harmful and causal effects of ROS, while the other supports beneficial effects. Recent studies have shown that interaction between ROS in different sub-cellular compartments plays a crucial role in determining the outcomes (beneficial vs. deleterious) of ROS exposures on the vascular system. Oxidant radicals in one cellular organelle can affect the ROS content and function in other sub-cellular compartments in endothelial cells (ECs). In this review, we will focus on a critical fact that the effects or the final phenotypic outcome of ROS exposure to EC are tissue- or organ-specific, and depend on the spatial (subcellular localization) and temporal (duration of ROS exposure) modulation of ROS levels.

## 1. ROS Paradox

Reactive oxygen species (ROS) have been associated with the pathogenesis of several diseases including cardiovascular disease. The presence of increased amount of ROS have been found to cause damages to major macromolecules in cells including vascular endothelial cell (EC) and other cell types in the cardiovascular system, resulting in cellular and systemic dysfunction. For example, endothelial dysfunction caused by increased ROS has been shown to contribute to the development of atherosclerosis, hypertension, ischemic heart disease, ischemic-reperfusion injury, and other vascular diseases. ROS have been reported to affect almost all organs in the body leading to pathologic conditions, such as pulmonary fibrosis and vascular retinopathy. Many studies have suggested a crucial role of antioxidants in the improvement of pathophysiological conditions. However, there is scarcity of data to support this notion in the clinics; instead, several studies failed to support this notion in the clinics. For instance, the multicenter clinical trial Heart Outcomes Prevention Evaluation (HOPE), which was carried out using antioxidants for several years, failed to produce any beneficial effects in the treatment of patients with cardiovascular disease [1]. Several studies from different groups demonstrated that a reduction in ROS had rather deleterious effects on cardiovascular system, including on endothelial cells [2,3]. Several recent studies supported a beneficial role of increased ROS in the vascular system depending on the sources and the duration of the subcellular ROS [4,5,6,7]. Taken together, although the above findings appear to support opposing roles of ROS (good or bad) in the cardiovascular pathophysiology, it suggests that the field of ROS biology lacks a clear understanding of the roles of ROS in physiological and pathological conditions in the body. In this review, we would like to propose that the beneficial versus harmful effects of the oxidants may not be understood using a reductionist’s linear thought process; instead, it may be an issue of ROS generation site, localization, amount, and duration of exposure. 

In the following paragraphs, we will discuss some of the harmful effects followed by the beneficial effects of the ROS that have been implicated in several major pathophysiological processes.

## 2. Reactive Oxygen Species (ROS)

**Figure 1:**
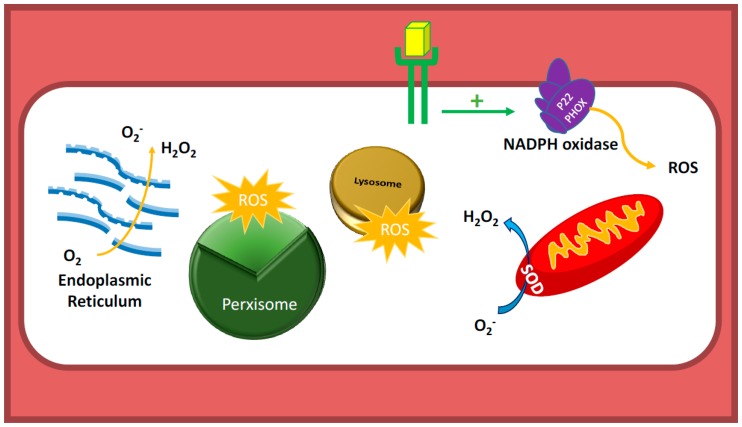
Sources of reactive oxygen species (ROS) in endothelial cells (EC). Nicotinamide Adenine Dinucleotide Phosphate (NADPH) oxidases are the major sources of ROS in ECs. Unlike in other cell types, mitochondria, which constitute only 5% of endothelial cell mass, are not believed to be major sources of ROS, as ECs do not depend on mitochondrial oxidative phosphorylation as their energy source. ECs, like tumor cells, utilize glycolysis as their source for Adenosine triphosphate (ATP) generation. Peroxisome, lysosome, and Endoplasmic Reticulum (ER) also produce ROS in EC. SOD: superoxide dismutase; P22 PHOX: P22 Phagocyte Oxidase.

There are several intracellular enzymes that produce oxidants, or ROS. ROS are molecules containing one or more unpaired electrons in their orbital that renders a considerable degree of reactivity to ROS. These unpaired electrons initiate a chain reaction by stealing electrons from other molecules to complete their orbital and become stable. Few examples are superoxide anion (O_2_^−^) and hydroxyl radical (^•^OH). However, there are other molecules that are “reactive”, but do not possess unpaired electrons, such as non-radical molecules like hydrogen peroxide (H_2_O_2_), peroxynitrite (ONOO^•^), and other oxidative molecules (Figure 1) [4,5,6,7].

There are several different intracellular sources for ROS, including Dinucleotide Phosphate (NADPH) oxidases, mitochondria, peroxisomes, lysosomes, xanthine oxidases, cytochrome P450, etc. (Figure 1) [4,5,6,7,8,9,10,11,12,13,14]. Initially ROS producing enzymes were discovered in phagocytic cells where numerous amount of ROS are produced physiologically by membrane-bound NADPH oxidase (NOX) enzymes to kill microbes. Subsequently, NOX was detected in several different cell types including vascular cells and cardiomyocytes, suggesting its involvement in the physiological processes. Although there are many different sources of ROS in the vasculature, NOX enzymes appear to be the major sources of ROS in physiological and pathological conditions. There are several different isoforms of NOX and, among them, the major contributors of ROS production in the vasculature are, namely, NOX1, NOX2 (gp91^phox^), NOX4, and NOX5. The Rac1 containing membrane-bound enzyme complex NADPH oxidase is composed of five subunits, two of which are membrane bound including Nox2 (gp91^phox^) and p22^phox^. The other three are regulatory cytoplasmic subunits, and they include p47^phox^, p67 ^phox^, and the Rac1 subunit that contains GTPase activity [8]. Recent studies have shown to have roles for NOX2 and NOX4 in endothelial cell dysfunction or survival depending on their subcellular localization and duration of activation. 

**Figure 2:**
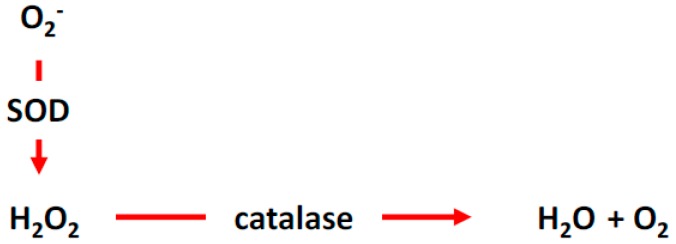
ROS cycle and homeostasis. Superoxide is converted the hydrogen peroxide, which is then catalyzed by catalase to water and molecular oxygen. SOD, superoxide dismutase.

NOX2 and NOX4 are shown to exist in the cell membrane and diverse subcellular compartments, such as the perinuclear membrane and endoplasmic reticulum [9,10]. As mentioned earlier, NOX2 enzyme generates ROS, specifically O_2_^−^, which may be converted to H_2_O_2_ in the presence of superoxide dismutases (SODs), and H_2_O_2_ are then converted to H_2_O and molecular O_2_ by catalases (Figure 2). Interestingly, whereas generation of ROS by NOX2 require all of the membrane-bound and cytosolic factors, NOX4 requires p22^phox^ and predominantly produces H_2_O_2_ without requiring activating cytosolic factors [4,6,11,12,13,14]. Thereafter, elevation of endogenous ROS levels by NOX2 and NOX4 may require different stimuli and their resulting effects on the vascular system may differ significantly.

Although this article explores both harmful and beneficial effects of the oxidants on the cardiovascular system with a special emphasis on vascular endothelial cells, we have included the effects of ROS on other tissues to provide a general understanding of effects of ROS to the readership. In the following sections, we will first summarize the deleterious effects of ROS reported in the literature. 

### 2.1. Endothelial Dysfunction

Endothelial dysfunction is defined as the loss of endothelium-dependent relaxation of the smooth muscle layer of the blood vessels. It occurs mainly due to a decreased level of available NO (nitric oxide), which is the major effector molecule in smooth muscle relaxation through a cyclic guanosine monophosphate (cGMP)-mediated calcium regulation signaling pathway [15]. Endothelial dysfunction may result from increased ROS levels generated through several different mechanisms. First, high levels of superoxide may react with NO resulting in the formation of a deleterious free radical called peroxynitrite. In fact, the rate of formation of peroxynitrite from superoxide is three times faster than the neutralization of superoxide through dismutation reaction, supporting the notion that higher levels of superoxide formation during pathological conditions is harmful. Second, ROS can be produced through uncoupling of the eNOS enzyme, the very enzyme that produces the beneficial signaling molecule NO under physiological conditions. Uncoupling of the enzyme may occur due to a deficiency in the enzyme substrate l-arginine, or the enzyme cofactor BH4 (tetrahydrobiopterin). As a result, eNOS may produce superoxide instead of NO, resulting in further depletion of NO [15]. Endothelial cell activation accompanies endothelial dysfunction, and is characterized by increased expression of adhesion molecules such as vascular cell adhesion molecule-1 (VCAM-1) and intercellular adhesion molecule-1 (ICAM-1). The upregulation of adhesion molecules enhances the interaction between inflammatory cells and the endothelium, resulting in the slowing down of neutrophil rolling and, subsequently, in their adhesion and transmigration into the sub-endothelial space. The inflammatory cells, such as neutrophils, also produce large amounts of ROS via activation of NADPH oxidase and myeloperoxidase during inflammation. However, during inflammation ROS play a significant beneficial role by helping the body to dispose of the pathogens, while, in other conditions, ROS may have harmful effects, such as in atherosclerosis and diabetic vasculopathy. 

**Figure 3:**
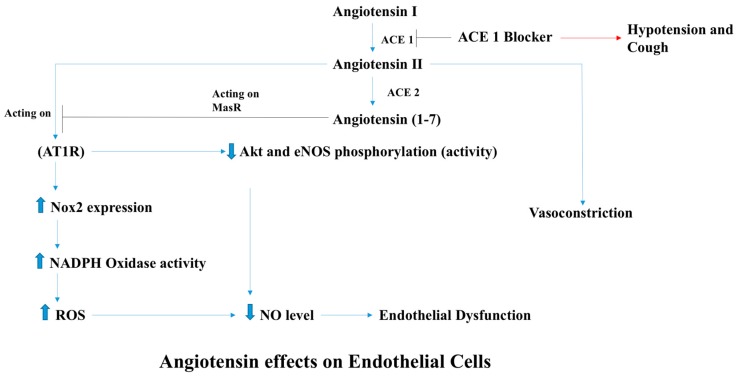
Effects of angiotensin on endothelial cells. AngII induces ROS production in endothelial cell, resulting in vasoconstriction. AngII plays a crucial role in the renin-angiotensin system that maintains blood pressure by inducing sodium and water retention, sympathetic activity, and potent vasoconstriction. Despite the fact that AngII is involved in the regulation of the blood pressure and tissue perfusion, it also exerts pathological effects that have been implicated in cardiovascular diseases, some of which are mediated through the activation of angiotensin type 1 receptor (AT1R). Nox2: NADPH Oxidase-2; ACE 1: Angiotensin Converting Enzyme-1; ACE 2: Angiotensin Converting Enzyme-2.

An angiotensin (Ang) II-infused animal model has been used as a model for ROS-induced cardiovascular disease including endothelial dysfunction leading to hypertension and vasculopathy. Although this model of AngII-ROS cannot exclude other confounding factors, several studies have demonstrated that AngII induces ROS production in endothelial cells, resulting in vasoconstriction [16,17,18,19]. AngII is an important effector molecule in the renin-angiotensin system that maintains blood pressure by inducing sodium and water retention, sympathetic activity, and potent vasoconstriction. Despite the fact that AngII is involved in the regulation of the blood pressure and tissue perfusion, it also exerts pathological effects that have been implicated in cardiovascular diseases, some of which are mediated through the activation of the angiotensin type 1 receptor (AT1R) (Figure 3) [20]. AngII has been shown to increase the production of ROS by augmenting NADPH oxidase activity and altering mitochondrial function using human brain microvascular EC (HbmECs), bovine aortic EC (BAEC), and human aortic EC (HAEC), [16,17,18,19,20,21,22]. As a consequence, AngII-derived ROS decelerate the activity of eNOS and decrease NO bioavailability, leading to endothelial dysfunction. 

### 2.2. Mitochondrial Dysfunction

**Figure 4:**
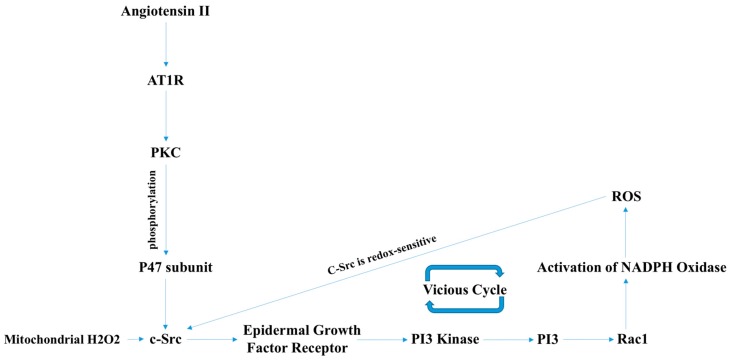
AngII induces NADPH oxidase-derived ROS and mitochondrial ROS, which, in turn, activate c-Src-PI3K-Rac1-induced NADPH oxidase, resulting in a positive feedforward “vicious cycle” of oxidative stress. H_2_O_2_ activates the c-Src enzyme, which, in turn, activates the Rac1-dependent NADPH oxidase enzyme and produces O_2_^−^. Activated c-Src induces the PI3K-Akt signaling cascade, which, in turn, activates Rac1 and, thus, NADPH oxidase, resulting in further ROS production. PKC: Protein Kinase-C.

AngII has also been reported to induce mitochondrial dysfunction [21]. AngII-induced mitochondrial dysfunction involves K^+^ (potassium ion) channel activation and leakage of K^+^ out of the mitochondria causing a drop in the mitochondrial membrane potential. K^+^ efflux also results in the opening of H/K antiporters leading to alkalization of the mitochondrial matrix. These findings of mitochondrial potassium leakage and alkalosis accompany concomitant ROS production mainly through RET (reverse electron transport) [19]. Mitochondrial ROS also have an influence on cytosolic ROS production by several different mechanisms, not all of which are studied well. For instance, H_2_O_2_ activates the c-Src enzyme which, in turn, activates the Rac1-dependent NADPH oxidase enzyme and produces O_2_^−^ (Figure 4) [16]. It is interesting to note that ROS scavengers targeted against both mitochondrial and cytosolic ROS resulted in decreased blood pressure in AngII-induced hypertension models, further supporting the notion that AngII-induced vascular pathologies involve increased levels of subcellular and cytosolic ROS [16,17,19].

### 2.3. Pulmonary and Renovascular Hypertension

**Figure 5:**
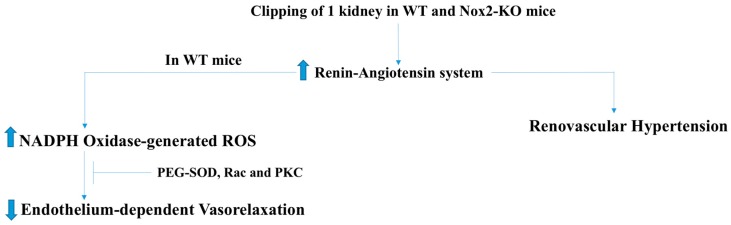
NADPH oxidase-derived ROS in renovascular hypetertension. Activation of PKC, Rac1, and tyrosine kinase receptors increases ROS production via activation of NADPH oxidase. ROS-induced inhibition of endothelium-dependent vasodilatation can be abrogated by inhibition of PKC, Rac, and EGFR kinase in renovascular animal model (using clipped WT mice), suggesting that activation of NADPH oxidase-derived ROS plays a crucial role in the development of renovascular hypertension. PEG-SOD: Polyethylene glycol-conjugated superoxide dismutase; WT: Wild type.

**Figure 6:**
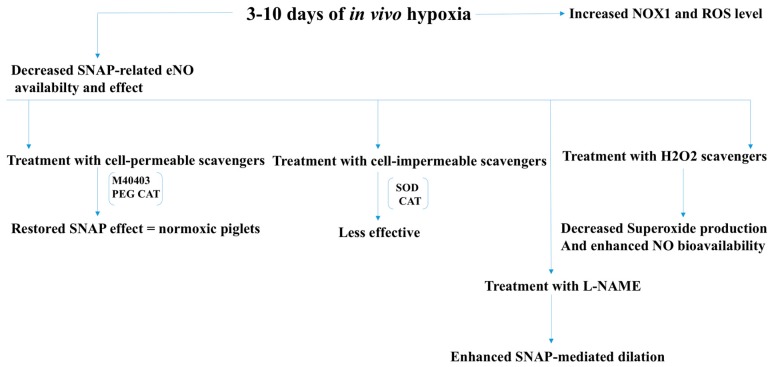
Increased NOX-derived ROS levels and eNOS uncoupling in hypoxic conditions give rise to pulmonary vasoconstriction resulting in pulmonary hypertension. ROS levels correlate with the level of hypoxia, which, in turn, play an important role in the pathogenesis and development of pulmonary hypertension. Combined treatment with superoxide-scavenger, H_2_O_2_-scavenger, and eNOS inhibitor (L-NAME) improve vasodilation in hypoxic conditions, suggesting that a hypoxia-induced increase in ROS levels involve both NOX activation and eNOS uncoupling. SNAP: Nitroso-N-acetyl-penicillamine; PEG CAT: Polyethylene glycol catalase; SOD: Superoxide dismutase; CAT: Catalase; NOX1: NADPH oxidase-1.

ROS have also been implicated in renovascular hypertension and chronic hypoxia-associated pulmonary hypertension. In renovascular hypertension, which is caused by increased renin release by the kidneys, ROS are believed to impair endothelium-dependent vasorelaxation [22]. The findings that in vivo administration of polyethylene glycol-conjugated superoxide dismutase (PEG-SOD), or in vitro treatment with antioxidant tiron improved endothelial function in an AngII animal model also supports a critical role of ROS. Activation of PKC, Rac1, and tyrosine kinase receptors is known to increase ROS production via activation of NADPH oxidase. Results showing inhibition of PKC, Rac, and endothelial growth factor receptor (EGFR) kinase restores endothelium-dependent vascular relaxation in a renovascular animal model (using clipped WT (wild type) mice), suggesting activation of NADPH oxidase in renovascular hypertension (Figure 5) [22]. Unlike other micro vessels in the body, pulmonary blood vessels constrict during hypoxia. It has been known for decades that cardiopulmonary disorders that are associated with hypoxia, such as congenital diaphragmatic hernia, lead to pulmonary hypertension with a survival rate for infants between 40% and 50% [23]. It has been found that ROS levels correlate with the level of hypoxia, which, in turn, play an important role in the pathogenesis and development of pulmonary hypertension. Interestingly, treatment of chronic pulmonary hypertension with inhaled NO, or NO donors, such as SNAP (*S*-nitroso-*N*-acetyl-penicillamine), as dilators for the pulmonary resistant arteries (PRAs) has failed [23]. Prompt conversion of NO to ONOO by increased ROS is considered to be a major cause behind this observation. This notion has been supported by the findings that the combined treatment with cell-permeable superoxide-scavenger M40403, and H_2_O_2_-scavenger polyethylene glycol catalase (PEG-CAT) restored the SNAP effect to the same level seen in pulmonary vessels of normoxic animals [23]. Moreover, treatment with eNOS inhibitor (L-NAME) enhanced SNAP-mediated dilation in hypoxic conditions suggesting that a hypoxia-induced increase in ROS levels involved eNOS uncoupling (Figure 6). 

### 2.4. Atherosclerosis and Ischemic Heart Disease

**Figure 7:**
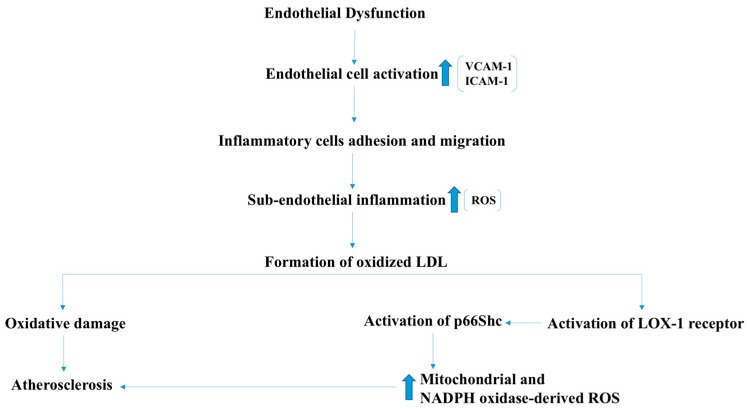
p66SHC plays crucial role in oxLDL-mediated oxidative damage to vascular endothelium through NADPH oxidase-derived and mitochondrial ROS. Initial damages to endothelium induces EC activation (vascular cell adhesion molecule-1 (VCAM1), intercellular adhesion molecule-1 (ICAM1) induction) and adhesion of inflammatory cells to EC resulting in ROS production. Inflammatory cell migration to sub-endothelial layer results in “foam call” formation. oxLDL-induced atherosclerotic changes involve inflammatory cells, activation of p66SHC and ROS from mitochondria and NADPH oxidase.

Endothelial dysfunction is one of the initial events that accompany atherosclerosis and ischemic Heart disease [24]. In atherosclerosis-predisposing conditions, such as hypercholesterolemia, ROS react with Low-density lipoproteins (LDL), resulting in the formation of oxidized LDLs (oxLDL), which are the major effectors in the development of atherosclerosis (Figure 7) [25]. In addition, oxLDLs were found to increase the formation of ROS by endothelial cells through the activation of LOX-1 receptor, and subsequent phosphorylation and activation of the p66Shc protein that regulates ROS production by the mitochondria (Figure 7) [26]. Genetic deletion of p66SHC encoding gene significantly reduced oxidant levels and atherosclerosis in apolipoproteinE-knock out (ApoE-KO) mice, suggesting an important role for the oxLDL-mediated oxidative stress in the development of atherosclerosis [27]. Additionally, suppression of the p66Shc expression decreased p47^phox^ subunit expression of the NADPH oxidase enzyme, which in turn decreased ROS generation. Together, these reports suggest that NADPH oxidase contributes to the oxLDL-induced ROS production by vascular cells (Figure 7) [28]. In atherosclerotic lesions, increased NADPH oxidase activity and high ROS levels have been reported. An increase in the expression of the p22phox subunit along with increased ROS levels in the atherosclerotic lesions of patients with unstable angina has been reported [29]. Furthermore, a significant decrease in the aortic ROS levels was observed in ApoE and p47^phox^-KO mice compared to ApoE-KO mice [30]. These results reveal the important role of NADPH oxidase in the development of atherosclerotic lesions. 

### 2.5. Ischemic-Reperfusion Injury

An ischemic injury to the myocardium causes hypoxia and decreased oxygenation to the area supplied by the stenosed or blocked coronary vessel. It is most commonly seen in atherosclerosis. The oxygen-deprived cells undergo a reversible cell injury if the lack of blood supply is transient or of short duration. Cardiac muscle cells are able to withstand ischemia for a maximum of 30 min, after which they undergo an irreversible injury followed by death by necrosis [31]. The necrotic tissue releases its cellular content, including enzymes and ROS into the surrounding environment. Reperfusion at this stage is more injurious than beneficial, a simplified account is that the spread of these toxic substances to the surroundings cause further damage to the surrounding healthy cells. However, there are several different pathways that involve increase generation of ROS and results in further injury during the early reperfusion stage. In animal models, glutathione peroxidase 1 deficiency has been shown to cause oxidative damage to the mitochondrial DNA and decrease the expression of the mitochondrial complex I protein, resulting in impaired NADH and Adenosine triphosphate (ATP) production by the ischemic myocardiocytes, leading to alterations in the metabolic state and loss of cellular viability [32]. 

### 2.6. Age-Related Macular Degeneration (AMD)

**Figure 8:**
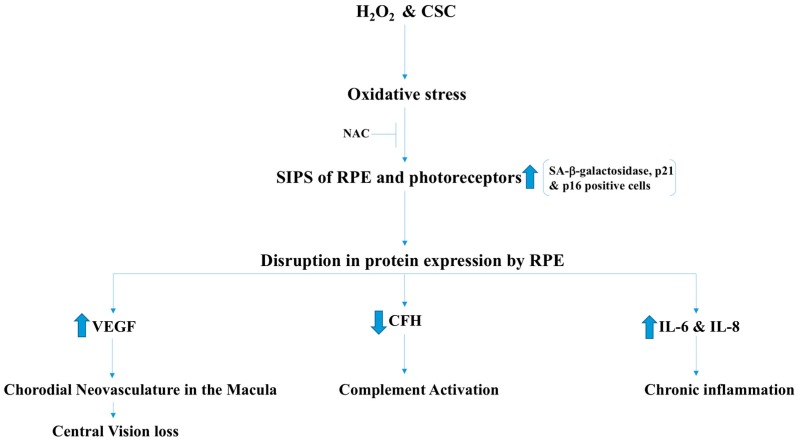
ROS-induced inflammation and damage to retinal pigmented epithelium (RPE) results in the activation of VEGF-induced choroidal neovasculature and Age-Related Macular Degeneration (AMD). Oxidative stress, including cigarette smoke and other inflammatory mediators, result in inflammatory damages to RPE, which, in turn, results in increase in vascular endothelial growth factor (VEGF), interlukine-6 (IL-6), interlukine-8 (IL-8), and activation of complement. These inflammatory mediators and endothelial growth factors induce a chronic inflammatory state including neovessel formation in the Macula, a hallmark of AMD. CSC: Cigarrete smoke concentrate; NAC: N-acetylcysteine; SIPS: stress-induced premature cellular senescence; CFH: complement factor H.

AMD is a degenerative retinal disease that causes blindness in people aged 60–65 years and older. AMD is characterized by the development of choroidal neovasculature (CNV) that affects the macula and damages the photoreceptor cells, leading to central vision loss. The development of CNV is due to the increased expression of vascular endothelial growth factor (VEGF) which was confirmed by Western blot and polymerase chain reaction (PCR) [33]. The disease involves degeneration of retinal pigmented epithelial cells (RPE) and the photoreceptors [33]. The most important risk factors include, smoking, age, and disruption in the expression of certain proteins. Stress-induced cellular senescence (SICS) contributes to this condition in which oxidative stress triggers senescence in RPE cells and leads to dysregulation in the expression of major proteins that contribute to the development of AMD. Cigarette smoke concentrate (CSC) is believed to contribute to the development of cellular senescence by supplying exogenous oxidant radicals [33]. Using human RPE cell line (ARPE-19), CSC was also found to cause oxidative DNA damage, leading to premature senescence that was evident by the high number of positive senescence-associated (SA) β-galactosidase, p21, and p16 cells. Senescent cells were found to upregulate the expression of the VEGF, which is the major stimulator of angiogenesis. VEGF and complement factor H (CFH) are major players in the development of AMD. Senescent cells also secrete pro-inflammatory IL-6 and IL-8 and induce chronic inflammation. Involvement of ROS in the induction of the senescence state was confirmed by reversing senescence using co-administration of N-acetylcysteine NAC (ROS scavenger) with CSC and H_2_O_2_ (Figure 8).

Despite the fact that the focus of this review was mainly on vascular endothelial and other cardiovascular tissues, other tissues in which ROS are produced have also been discussed. For instance, oxidative stress is known as a major factor in the development of AMD. It must be noted that oxidative stress induces cellular senescence, that causes alterations in gene expression, most importantly the expression of VEGF, which is the major effector molecule in the development of the choroidal microvasculature in the macula, leading to central vision loss (Figure 8). Although angiogenesis is required for overcoming an ischemic attack in the heart, aberrant angiogenesis is harmful in other sites, such as the retina. 

## 3. Beneficial Effects of Sub-Cellular ROS

Since the discovery of ROS, researchers have postulated their harmful effects as their levels were drastically increased in cardiovascular pathology, which counts as one of the primary causes of mortality worldwide. Association or co-existence of increased levels of ROS have been established during the progression of cardiovascular diseases. Several researchers hypothesized that halting abnormal ROS production and augmenting the innate antioxidants protective activity may result in beneficial outcomes in the treatment of cardiovascular diseases. However, in the multi-center, large clinical trials, such as HOPE, a surprising failure to improve the condition of the cardiovascular disease (CVD) patients left researchers intrigued [1]. Other studies have postulated that reduction in ROS have a further deleterious effect in cardiovascular problems [2,3]. Thus, a novel hypothesis has been developed by a few researchers regarding the paradoxical effects of ROS in cardiovascular diseases. Several recent studies reported beneficial roles of increased ROS derived from NOX2, NOX4, and mitochondria in vascular cells depending on the duration of ROS exposure and their (ROS) sources and localization. 

**Figure 9:**
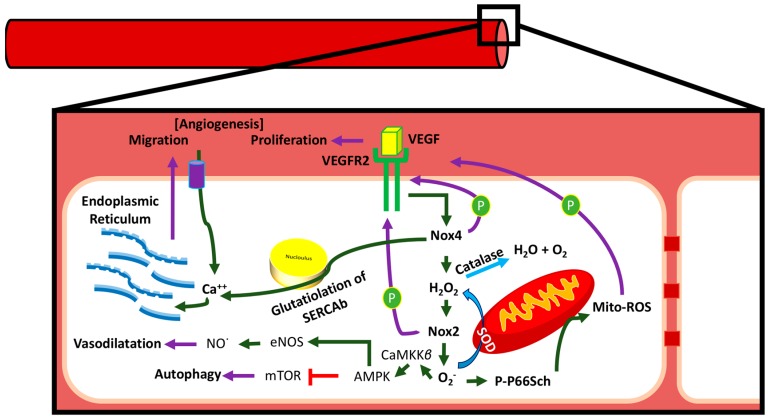
NADPH oxidase and NOX4 play major roles in endothelial ROS production and signal transduction. Endothelial ROS induces activation of eNOS to improve nitrogen oxide- cyclic guanine monophosphate (NO-cGMP)-mediated vasorelaxation, AMP-activated protein kinase- mammalian target of rapamycin (AMPK-mTOR)-mediated autophagy, glutathiolation of SERCAb-induced Ca^++^ influx in ER and migration, and p66Shc-mediated modulation of mitochondrial ROS. Activation of VEGFR2 by VEGF leads to the activation of Nox4 and NADPH oxidase, resulting in intracellular redox signal activation. AMPK: AMP-activated protein kinase; CaMKKβ: Ca2+/Calmodulin-dependent protein kinase kinase-beta; PP66Sch: phosphorylated-P66Sch.

**Figure 10:**
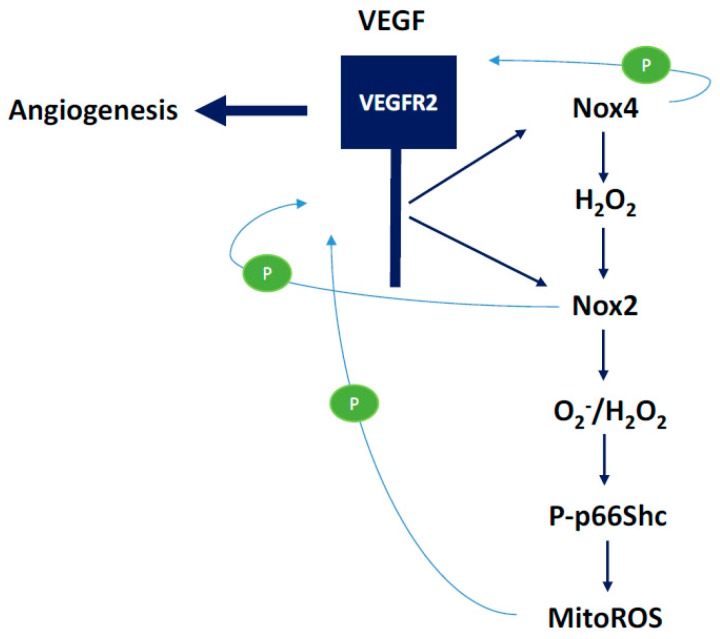
The role of Nox2- and NOX4-derived ROS in the activation of the p66shc-mediated modulation of mitochondrial ROS. VEGF-mediated angiogenic pathways involve ROS-mediated activation of endothelial cell signaling cascades. Nox2 and Nox4-derived ROS have been shown to induce increased expression and activation of VEGFR2, the major endothelial receptor involved in cell signaling leading to pro-survival, growth, and angiogenic signals.

**Figure 11:**
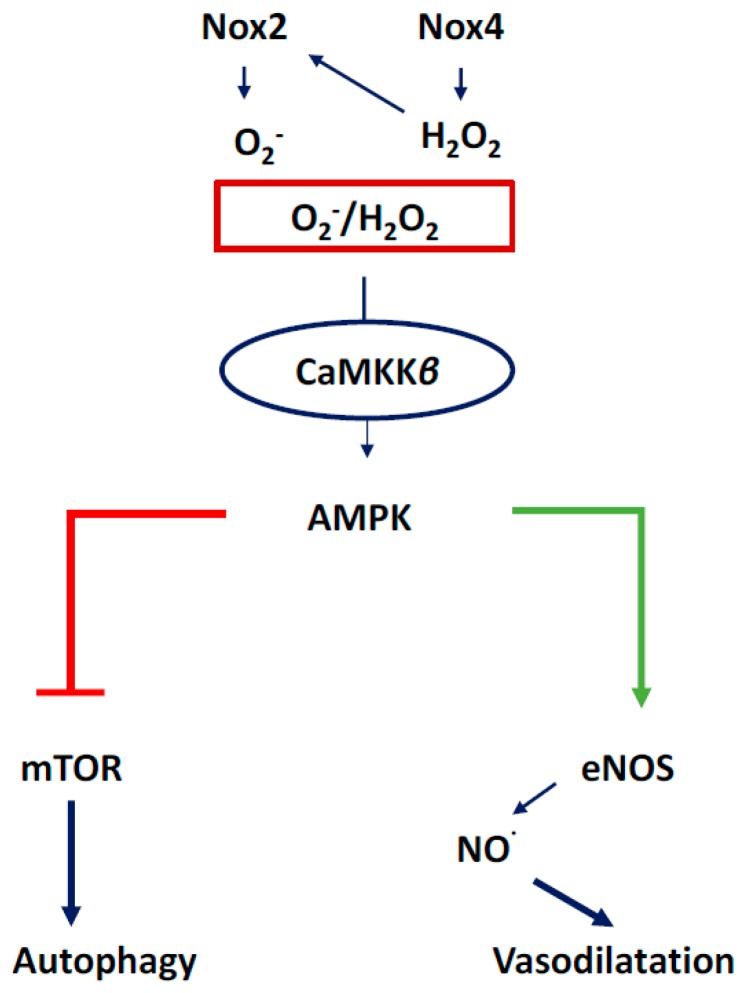
ROS-induced activation of CaMKKβ, AMPK, and downstream activation of eNOS and the inhibition of mTOR. AMPK-mediated activation of eNOS results in NO-mediated vasodilation of coronary vessels, while AMPK-mediated inhibition of mTOR induces autophagy to dispose of cellular debris and recycle sub-cellular components for cell survival.

**Figure 12:**
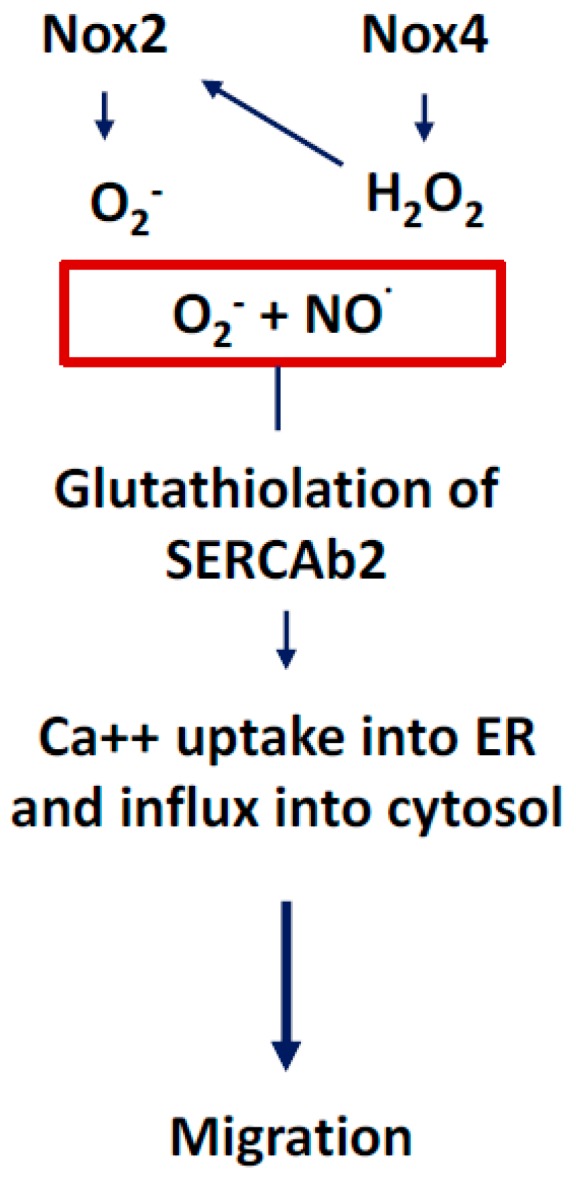
ROS-induced glutathiolation of SERCAb2 mediates endoplasmic reticulum (ER) Ca^++^ uptake, which, in turn, results in endothelial cell migration, an important cellular function involved in angiogenesis. Both O_2_^−^ and NO· are involved in the activation of signaling cascades that phosphorylate sarcoplasmic/endoplasmic reticulum calcium ATPase-b2 (SERCAb2) to increase the influx of Ca^++^ into the cytosol and endoplasmic reticulum, stimulating endothelial cell migration.

We will discuss the beneficial effects of ROS in this section with an emphasis on NADPH oxidase-derived oxidants. Activation of NOX2 and NOX4 to produce ROS has been shown to induce activation of intracellular signaling pathways that are essential for physiological functions of cardiovascular system including ECs (Figure 9). There are several intracellular and extracellular stimuli that can mediate NOX-derived ROS generation. VEGF is a major activating factor for both NOX2 and NOX4 to induce angiogenesis and other essential functions of ECs, such as NO synthesis and hemostasis [2]. VEGF binds to VEGF receptors on EC. Vascular endothelial growth factor receptor-2 (VEGFR2) is a major tyrosine kinase receptor that activates NOX enzymes including NOX2 and NOX4 via different signaling pathways in EC (Figure 9). Interestingly, a NOX4-mediated increase in H_2_O_2_ has been shown to increase the expression of NOX2 and, thus, an increase in O_2_^−^ (Figure 10), suggesting a critical communication between different NOX enzymes [4]. Both H_2_O_2_ and O_2_^−^ are implicated in cell survival through different pathways in physiological and also in pathological conditions (Figure 10). For example, O_2_^−^ has been demonstrated to phosphorylate AMP-activated protein kinase (AMPK) via Ca2+/Calmodulin-dependent protein kinase kinase-beta (CaMKKβ) to induce eNOS phosphorylation and synthesis of NO resulting in vasorelaxation, and to decrease the level of mammalian target of rapamycin (mTOR) activity resulting in a protective autophagy response (Figure 11) [6]. Both O_2_^−^ and NO phosphorylate sarcoplasmic/endoplasmic reticulum calcium ATPase-b2 (SERCAb2) to increase the influx of Ca^++^ into the cytosol and endoplasmic reticulum, stimulating endothelial cell migration (Figure 12) [34]. NOX-derived ROS have been shown to play important roles in regulating mitochondrial oxidant levels through NOX2-NOX4-derived ROS-mediated Ser36-p66Shc-phosphorylation that stimulates mitochondrial ROS production (Figure 10) [4]. Additionally, ROS produced by NOX2, NOX4, and mitochondria induce VEGFR2 activation, resulting in angiogenesis [4].

### 3.1. NOX2-Containing NADPH Oxidase

Nox2 or gp91^phox^, a major component of NADPH oxidase, has been shown to play a positive role in endothelial function using binary conditional transgenic mice [6]. This study demonstrated that an EC-specific increase in NOX2 expression resulted in increasing ROS production in an endothelium-specific manner, which, in turn, activated AMPK-eNOS signaling pathways and resulted in improved coronary vasorelaxation, EC proliferation, and aortic sprouting ex vivo (Figure 11). Inhibition of NADPH oxidase activity in human coronary artery EC (HCAEC), pulmonary artery EC (HPAEC), and bovine aortic artery EC (BAEC) resulted in inhibition of EC proliferation and migration [2]. However, in in vitro cell culture assays using HCAEC, NADPH oxidase-derived ROS were shown to increase eNOS activity and NO synthesis via activation of the phosphoinositide 3-kinase (PI3K)-protein kinase B (Akt)-endothelial nitric oxide synthase (eNOS) signaling pathway [8,35]. Interestingly, increased NOX-derived cytosolic ROS levels were found to induce increased ROS in mitochondria, which was counteracted by increased levels and activity of the mitochondrial antioxidant MnSOD in transgenic animals with increased EC-specific NOX2 expression [6]. Whereas NOX-derived ROS have been shown to play major roles in VEGFR2 receptor activation, NOX4 knockout mice did not demonstrate a major effect on VEGFR2 activity. These findings suggest NOX2 plays a major role in ROS-induced angiogenesis [4]. 

### 3.2. NOX4

**Figure 13:**
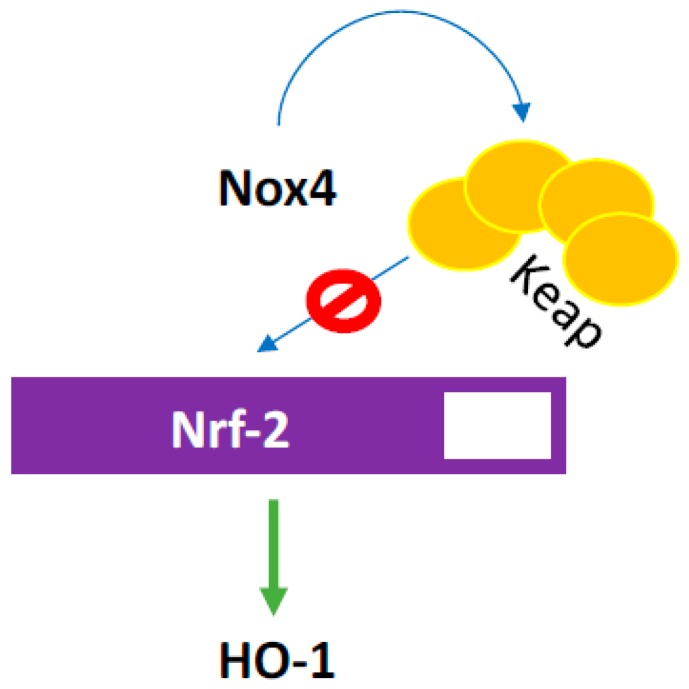
ROS induces Keap oxidation which, in turn, prevents degradation of Nrf-2 transcription factor that increases hemoxygenase 1 (HO-1) expression. HO, a critical enzyme that performs several critical functions within vascular cells including heme degradation and, in turn releasing bile pigments and carbon monoxide, has significant antioxidant and signaling effects in its active and inactive forms.

Several studies have reported various beneficial effects of Nox4-derived H_2_O_2_ both in vitro and in vivo. First, H_2_O_2_ stimulates endothelium-dependent vasodilatation through hyperpolarization and plays an important role in maintaining blood pressure in Angiotensin II-infused animal model [14]. Hypoxia increases NOX4-derived H_2_O_2_ levels and H_2_O_2_, which, in turn, activates transforming growth factor β1 (TGFβ1), resulting in increased sprouting angiogenesis, acceleration of blood flow, and an increase in hemoglobin content [7]. NOX4 also inhibited activation of the apoptotic caspases resulting in inhibition of apoptosis and improvement in cell survival [7]. Several studies demonstrated that reduction in HO-1 was accompanied by an increase in apoptosis and endothelial E-selectin expression in NOX4-knockout mice. These studies demonstrated that NOX4 supports Keap oxidation, which, in turn, prevents Nrf-2 degradation, a transcription factor that regulates HO-1 expression (Figure 13) [5]. HO, a critical enzyme that performs several critical functions within vascular cells, including heme degradation and, in turn, releasing bile pigments and carbon monoxide, has significant antioxidant and signaling effects in its active and inactive forms. 

### 3.3. Mitochondrial ROS

Mitochondrial oxidants play crucial roles in cellular biology, especially in cardiovascular cell signaling mechanisms, of which very little is known. Recent evidence supports important EC functions are modulated by mitochondrial ROS. There are several recent studies that also show a correlation and subcellular communication between the endogenous cytosolic ROS and mitochondrial ROS [4,10]. Few have claimed that an increase in cytosolic ROS in the short-term can protect the vascular cells by inducing mitochondrial ROS scavengers, such as MnSOD [10]. As mentioned earlier, mitochondrial oxidants can also induce phosphorylation of VEGFR2, resulting in angiogenesis (Figure 10) [33]. In contrast to the exposure in the short-term, ROS induces uncontrolled levels of mitochondrial oxidants in the vascular endothelium [10]. Inactivation of MnSOD activity by nitro-tyrosine modification due to increased levels of peroxynitrite (ONOO^•^) during long-term exposure to NOX-ROS was shown to be responsible for this temporal effect. These findings suggested accumulation of ONOO^•^ plays a critical role in determining the effects of ROS (beneficial versus deleterious) on endothelial phenotype. The apparent paradox of ROS effects on endothelium reported will be better understood by carefully studying both duration of exposure (temporal) and subcellular localizations (spatial) of ROS. It is to be noted that due to recent developments of tools for studying mitochondrial biology, novel findings about mitochondrial ROS and their spatial and temporal roles are being investigated elaborately. An elegant study using cardiac-specific Trx2 knockout mice by Huang et al. [36] recently demonstrated that suppression of mitochondrial ROS can preserve cardiac function by inhibiting apoptosis signal regulating kinase-1 (ASK1)-mediated apoptosis. However, further studies are required to understand the roles of mitochondrial ROS in the coronary vascular endothelium during myocardial ischemia or other cardiovascular pathologies. 

### 3.4. Communication between Sub-Cellular ROS 

Several studies has shown that NADPH oxidase-derived and Nox4-derived ROS play crucial roles in growth factors- and hypoxia-induced angiogenesis including coronary angiogenesis [2,6,35,37,38,39]. NADPH oxidase-derived ROS have been shown to activate selective signal transduction cascades in endothelial cells such as PI3K-Akt-eNOS and AMPK-eNOS pathways to induce EC proliferation. Communication between sub-cellular ROS, such as between cytosolic (e.g., NADPH oxidase) and mitochondrial ROS, has been shown to have significant effects on endothelial function and coronary angiogenesis using ex vivo microvessel reactivity and aortic sprouting assays, respectively [6,10]. Together, these reports suggested that endothelial phenotype and function depend on a critical balance between sub-cellular ROS levels. 

## 4. Conclusions

In conclusion, we have addressed two opposing perspectives regarding ROS and their roles in the cardiovascular system. In cardiovascular diseases (CVD), especially ischemic heart disease (IHD), the effect of ROS remains controversial and requires further investigation. Recent studies have shown interaction between ROS in different sub-cellular compartments plays a crucial role in determining the outcomes (beneficial vs. deleterious) of ROS exposures on the vascular system. Oxidant radicals in one cellular organelle can affect the ROS content and function in other sub-cellular compartments [40]. For instance, an increase in NOX-derived ROS was found to have increased mitochondrial ROS and metabolism of vascular ECs. Mitochondrial ROS were also found to play a critical role in the development of cardiovascular diseases by increasing ROS content of the cytosol. Recent reports have claimed that the short-term exposure (four to eight weeks) to supra-physiological levels of NOX-ROS has beneficial effects such increase in EC proliferation and vascular sprouting [10]. However, these studies cannot conclude whether these beneficial effects were brought about simply by increased NOX-ROS or mito-ROS play a positive role since an increase in mito-ROS was also observed in these ECs. Interestingly, it was found that prolonged exposure to high levels of NOX-ROS impair endothelium-dependent vasodilation, which was accompanied by a corresponding increase in mito-ROS. Studies aimed at specifically reducing mitochondrial ROS while keeping NOX-ROS elevated may elucidate the roles of subcellular ROS in cardiovascular pathophysiology.

The linear query of whether ROS are harmful or beneficial is not well-formulated. Several studies demonstrated the harmful effects of ROS, while others found that they also have beneficial effects. In the current review, we pointed out a critical fact that the effects or the final phenotypic outcome of ROS exposure to EC are tissue- or organ-specific, and depend on the spatial (subcellular localization) and temporal (duration of ROS exposure) modulation of ROS. Thus, a simplistic, linear, or reductionist approach will not be able to elucidate the effects of ROS on the EC phenotype; rather, a holistic perspective with a systematic approach is essential to understand the spatiotemporal roles for ROS in angiogenesis and cardiovascular pathophysiology.
